# Demonstration of a near-IR line-referenced electro-optical laser frequency comb for precision radial velocity measurements in astronomy

**DOI:** 10.1038/ncomms10436

**Published:** 2016-01-27

**Authors:** X. Yi, K. Vahala, J. Li, S. Diddams, G. Ycas, P. Plavchan, S. Leifer, J. Sandhu, G. Vasisht, P. Chen, P. Gao, J. Gagne, E. Furlan, M. Bottom, E. C. Martin, M. P. Fitzgerald, G. Doppmann, C. Beichman

**Affiliations:** 1 Department of Applied Physics and Materials Science, Pasadena, California 91125, USA; 2 National Institute of Standards and Technology, 325 Broadway, Boulder, Colorado 80305, USA; 3 Department of Physics, University of Colorado, 2000 Colorado Avenue, Boulder, Colorado 80309, USA; 4 Department of Physics, Missouri State University, 901 S National Avenue, Springfield, Missouri 65897, USA; 5 Jet Propulsion Laboratory, California Institute of Technology, 4800 Oak Grove Drive, Pasadena, California 91109, USA; 6 Division of Geological and Planetary Sciences, California Institute of Technology, Pasadena, California 91125, USA; 7 Department of Terrestrial Magnetism, Carnegie Institution of Washington, 5241 Broad Branch Road, Washington, District of Columbia 20015, USA; 8 NASA Exoplanet Science Institute, California Institute of Technology, Pasadena, California 91125, USA; 9 Department of Astronomy, California Institute of Technology, Pasadena, California 91125, USA; 10 Department of Physics and Astronomy, University of California Los Angeles, Los Angeles, California 90095, USA; 11 W.M. Keck Observatory, Kamuela, Hawaii 96743, USA

## Abstract

An important technique for discovering and characterizing planets beyond our solar system relies upon measurement of weak Doppler shifts in the spectra of host stars induced by the influence of orbiting planets. A recent advance has been the introduction of optical frequency combs as frequency references. Frequency combs produce a series of equally spaced reference frequencies and they offer extreme accuracy and spectral grasp that can potentially revolutionize exoplanet detection. Here we demonstrate a laser frequency comb using an alternate comb generation method based on electro-optical modulation, with the comb centre wavelength stabilized to a molecular or atomic reference. In contrast to mode-locked combs, the line spacing is readily resolvable using typical astronomical grating spectrographs. Built using commercial off-the-shelf components, the instrument is relatively simple and reliable. Proof of concept experiments operated at near-infrared wavelengths were carried out at the NASA Infrared Telescope Facility and the Keck-II telescope.

## Abstract (web summary)

Laser frequency combs emit a spectrum of equally spaced peaks that can provide precise frequency references useful for astronomy. Here, the authors demonstrate a frequency comb using electro-optical modulation, which has a line spacing that is resolvable using grating spectrographs unlike the mode-locking approach.

## Graphical abstract

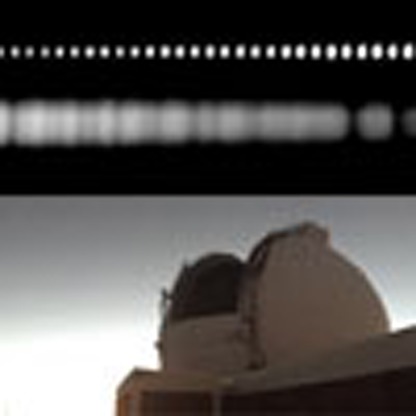

The earliest technique for the discovery and characterization of planets orbiting other stars (exoplanets) is the Doppler or radial velocity (RV) method whereby small periodic changes in the motion of a star orbited by a planet are detected via careful spectroscopic measurements^1^. The RV technique has identified hundreds of planets ranging in mass from a few times the mass of Jupiter to less than an Earth mass, and in orbital periods from less than a day to over 10 years (ref. 2). However, the detection of Earth-analogues at orbital separations suitable for the presence of liquid water at the planet's surface, that is, in the ‘habitable zone'^3^, remains challenging for stars like the Sun with RV signatures <0.1 m s^−1^ (Δ*V*/*c*<3 × 10^−10^) and periods of a year (∼10^8^ sec to measure three complete periods). For cooler, lower luminosity stars (spectral class M), however, the habitable zone moves closer to the star which, by application of Kepler's laws, implies that a planet's RV signature increases, ∼0.5 m s^−1^ (Δ*V*/*c*<1.5 × 10^−9^), and its orbital period decreases, ∼30 days (∼10^7^ s to measure three periods). Both of these effects make the detection easier. But for M stars, the bulk of the radiation shifts from the visible wavelengths, where most RV measurements have been made to date, into the near-infrared. Thus, there is considerable interest among astronomers in developing precise RV capabilities at longer wavelengths.

Critical to precision RV measurements is a highly stable wavelength reference^4^. Recently a number of groups have undertaken to provide a broadband calibration standard that consists of a ‘comb' of evenly spaced laser lines accurately anchored to a stable frequency standard and injected directly into the spectrometer along with the stellar spectrum^5^,^6^,^7^,^8^,^9^. While this effort has mostly been focused on visible wavelengths, there have been successful efforts at near-IR wavelengths as well^10^,^11^,^12^. In all of these earlier studies, the comb has been based on a femtosecond mode-locked laser that is self-referenced^13^,^14^,^15^, such that the spectral line spacing and common offset frequency of all lines are both locked to a radio frequency standard. Thus, laser combs potentially represent an ideal tool for spectroscopic and RV measurements.

However, in the case of mode-locked laser combs, the line spacing is typically in the range of 0.1–1 GHz, which is too small to be resolved by most astronomical spectrographs. As a result, the output spectrum of the comb must be spectrally filtered to create a calibration grid spaced by >10 GHz, which is more commensurate with the resolving power of a high-resolution astronomical spectrograph^8^. While this approach has led to spectrograph characterization at the cm s^−1^ level^16^, it nonetheless increases the complexity and cost of the system.

In light of this, there is interest in developing photonic tools that possess many of the benefits of mode-locked laser combs, but that might be simpler, less expensive and more amenable to ‘hands-off' operation at remote telescope sites. Indeed, in many RV measurements, other system-induced errors and uncertainties can limit the achievable precision, such that a frequency comb of lesser precision could still be equally valuable. For example, one alternative technique recently reported is to use a series of spectroscopic peaks induced in a broad continuum spectrum using a compact Fabry–Perot interferometer^17^,^18^,^19^. While the technique must account for temperature-induced tuning of the interferometer, it has the advantage of simplicity and low cost. Another interesting alternative is the so-called Kerr comb or microcomb, which has the distinct advantage of directly providing a comb with spacing in the range of 10–100 GHz, without the need for filtering^20^. While this new type of laser comb is still under development, there have been promising demonstrations of full microcomb frequency control^21^,^22^ and in the future it could be possible to fully integrate such a microcomb on only a few square centimetres of silicon, making a very robust and inexpensive calibrator. Another approach that has been proposed is to create a comb through electro-optical modulation of a frequency-stabilized laser^23^,^24^.

In the following, we describe a successful effort to implement this approach. We produce a line-referenced, electro-optical modulation frequency comb (LR-EOFC) ∼1559.9 nm in the astronomical H band (1,500–1,800 nm). We discuss the experimental set-up, laboratory results and proof of concept demonstrations at the NASA Infrared Telescope Facility (IRTF) and the W. M. Keck observatory (Keck) 10 m telescope.

## Results

### Comb generation

A LR-EOFC is a spectrum of lines generated by electro-optical modulation of a continuous-wave laser source^25^,^26^,^27^,^28^,^29^ which has been stabilized to a molecular or atomic reference (for example, *f*_0_=*f*_atom_). The position of the comb teeth (*f*_*N*_=*f*_0_±*Nf*_m_, *N* is an integer) has uncertainty determined by the stabilization of *f*_0_ and the microwave source that provides the modulation frequency *f*_m_. However, the typical uncertainty of a microwave source can be sub-Hertz when synchronized with a compact Rb clock and moreover can be global positioning system (GPS)-disciplined to provide long-term stability^12^. Thus, the dominant uncertainty in comb tooth frequency in the LR-EOFC is that of *f*_0_.

**Figure 1:**
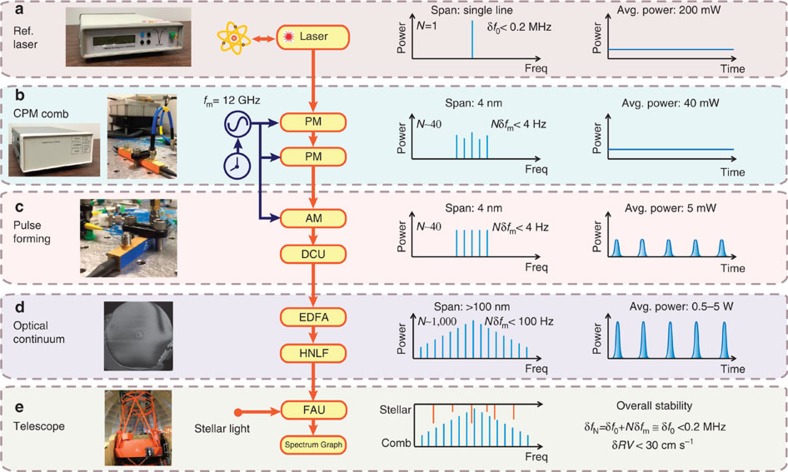
Conceptual schematics of the line-referenced electro-optical frequency comb for astronomy. Vertically, the first column contains images of key instruments. (**a**–**e**) The images are reference laser, Rb clock (left) and phase modulator (right), amplitude modulator, highly nonlinear fibre and telescope. A simplified schematic set-up is in the second column. Third and fourth columns present the comb state in the frequency and temporal domains. The frequency of *N*-th comb tooth is expressed as *f*_*N*_=*f*_0_+*N* × *f*_m_, where *f*_0_ and *f*_m_ are the reference laser frequency and modulation frequency, respectively. *N* is the number of comb lines relative to the reference laser (taken as comb line *N*=0), *RV* is radial velocity and *δf_N_*, *δf*_0_ and *δf*_m_ are the variance of *f*_N_, *f*_0_ and *f*_m_. (**a**) The reference laser is locked to a molecular transition, acquiring stability of 0.2 MHz, corresponding to 30 cm s^−1^ *RV*. (**b**) Cascaded phase modulation (CPM) comb: the phase of the reference laser is modulated by two phase modulators (PM), creating several tens of sidebands with spacing equal to the modulation frequency. The RF frequency generator is referenced to a Rb clock, providing stability at the sub-Hz level (*δf*_m_<0.03 Hz at 100 s). (**c**) Pulse forming is then performed by an amplitude modulator (AM) and dispersion compensation unit (DCU), which could be a long single mode fibre (SMF) or chirped fibre Bragg grating (FBG). (**d**) After amplification by an erbium-doped fibre amplifier (EDFA), the pulse undergoes optical continuum broadening in a highly nonlinear fibre (HNLF), extending its bandwidth >100 nm. (**e**) Finally the comb light is combined with stellar light using a fibre acquisition unit (FAU) and is sent into the telescope spectrograph. The overall comb stability is primarily determined by the pump laser.

**Figure 2:**
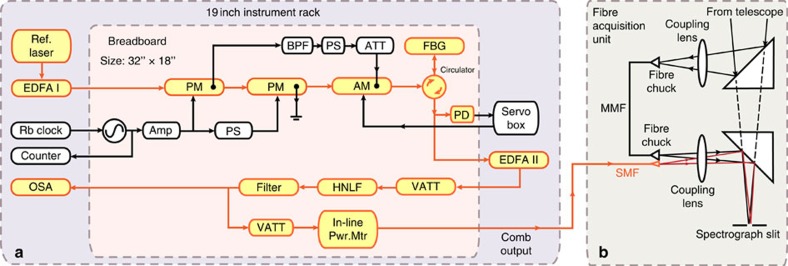
Detailed set-up of line-referenced electro-optical frequency comb. (**a**) The entire LR-EOFC system sits in a 19 inch instrument rack. Optics and microwave components in the rack are denoted in orange and black, respectively. Small components were assembled onto a breadboard. These included the phase modulators (PM), amplitude modulator (AM), fibre Bragg grating (FBG), photodetector (PD), variable attenuator (VATT), attenuator (ATT), highly nonlinear fibre (HNLF), microwave source, microwave amplifier (Amp), phase shifter (PS) and band-pass filter (BPS). The reference laser, erbium-doped fiber amplifier (EDFA), rubidium (Rb) clock, counter, optical spectrum analyser (OSA) and servo lock box are separately located in the instrument rack. (**b**) A simplified schematic of the fibre acquisition unit (FAU) is also shown. Stellar light is focused and coupled into a multimode fibre (MMF). The comb light from a single mode fibre (SMF), together with the stellar light in the MMF, are focused on the spectrograph slit and sent into the spectrograph.

**Figure 3:**
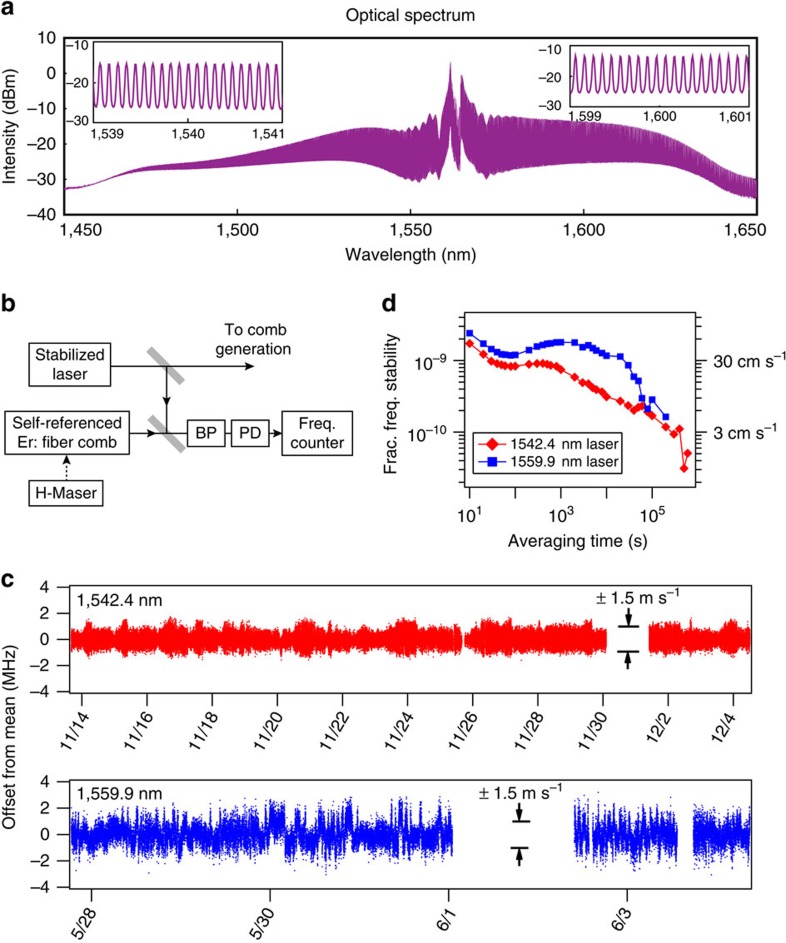
Comb spectra and stability of the C_2_H_2_ and HCN reference lasers. (**a**) A typical comb spectrum from the 1,559.9 nm laser with >100 nm span generated with 600 mW pump power. The insets show the resolved line spacing of 12 GHz or ∼0.1 nm. (**b**) Experimental set-up: BP, optical band-pass filter; PD, photodiode. All beam paths and beam combiners are in single mode fibre. (**c**) Time series of measured beat frequencies for the two frequency-stabilized lasers with 10 s averaging per measurement. The *x* axes are the dates in November of 2013 and May/June of 2014, respectively. (**d**) Allan deviation, which is a measure of the fractional frequency stability, computed from the time series data of **c**. Right-side scale gives the radial velocity precision.

The schematic layout for LR-EOFC generation is illustrated in Fig. 1 and a detailed layout is shown in Fig. 2. All components are commercially available off-the-shelf telecommunications components. Pictures of the key components are shown in the left column of Fig. 1. The frequency-stabilized laser is first pre-amplified to 200 mW with an Erbium-Doped Fibre Amplifier (EDFA, model: Amonics, AEDFA-PM-23-B-FA) and coupled into two tandem lithium niobate (LiNbO_3_) phase modulators (*V*_π_=3.9 V at 12 GHz, RF input limit: 33 dBm). The phase modulators are driven by an amplified 12 GHz frequency signal at 32.5 and 30.7 dBm, and synchronized by using microwave phase shifters. This initial phase modulation process produces a comb having ∼40 comb lines (≈2*π* × *V*_drive_/*V*_π_), or equivalently 4 nm bandwidth. This comb is then coupled into a LiNbO_3_ amplitude modulator with 18–20 dB distinction ratio, driven at the same microwave frequency by the microwave power recycled from the phase modulator external termination port. The modulation index of *π*/2 is set by an attenuator and the phase offset of the two amplitude modulator arms is set and locked to *π*/2. Microwave phase shifters are used to align the drive phase so that the amplitude modulator gates-out only those portions of the phase modulation that are approximately linearly chirped with one sign (that is, parabolic phase variation in time). A nearly transform-limited pulse is then formed when this parabolic phase variation is nullified by a dispersion compensation unit using a chirped fibre Bragg grating with 8 ps nm^−1^ dispersion. A 2 ps full-width at half-maximum pulse is measured after the fibre grating using an autocorrelator. Owing to this pulse formation, the duty cycle of the pulse train reaches below 2.5%, boosting the peak intensity of the pulses. These pulses are then amplified in a second EDFA (IPG Photonics, EAR-5 K-C-LP). For an average power of 1 W, peak power (pulse energy) is 40 W (83 pJ). The amplified pulses are then coupled into a 20 m length of highly nonlinear fibre with 0.25±0.15 ps nm^−1^ km^−1^ dispersion and dispersion slope of 0.006±0.004 ps nm^−2^ km^−1^. Propagation in the highly nonlinear fibre causes self-phase modulation and strong spectral broadening of the comb^30^. Comb spectra span and envelope can be controlled by the pump power launched into the highly nonlinear fibre. A typical comb spectrum with >600 mW pump power from the 1,559.9 nm laser is shown in Fig. 3a, with >100 nm spectral span. Moreover, by using various nonlinear fibre and spectral flattening methods, broad combs with level power are possible^31^.

**Figure 4:**
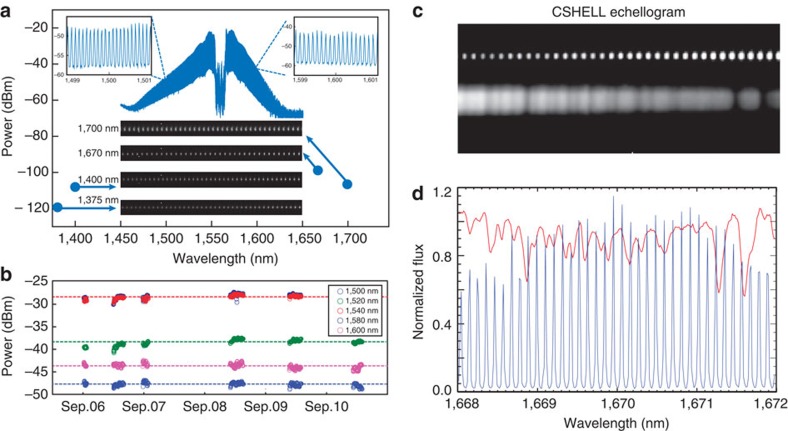
Experimental results at IRTF. (**a**) Comb spectrum produced using 1,559.9 nm reference laser. The insets on top left and right show the resolved comb lines on the optical spectrum analyser. Comb spectra taken by the CSHELL spectrograph at 1,375, 1,400, 1,670 and 1,700 nm are presented as insets in the lower half of the figure. The blue circles mark the estimated comb line power and centre wavelength for these spectra. Comb lines are detectable on CSHELL at fW power levels. (**b**) Comb spectral line power versus time is shown at five different wavelengths. During the 5 day test at IRTF, no parameter adjustment was made, and comb intensity was very stable even with multiple power-on and -off cycling of the optical continuum generation system. (**c**) An image of the echelle spectrum from CSHELL on IRTF showing a 4 nm portion of spectrum ∼1,670 nm. The top row of dots are the laser comb lines, while the broad spectrum at the bottom is from the bright M2 II–III giant star *β* Peg seen through dense cloud cover. (**d**) Spectra extracted from **c**. The solid red curve denotes the average of 11 individual spectra of *β* Peg (without the gas cell) obtained with CSHELL on the IRTF. The regular sine-wave like blue lines show the spectrum from the laser comb obtained simultaneously with the stellar spectrum. The vertical axis is normalized flux units.

The LR-EOFC system is mounted on an aluminum breadboard (18" × 32", or equivalently 45.7 × 81.3 cm) in a standard 19-inch instrument rack (see Fig. 2) for transport and implementation with the spectrograph at the NASA IRTF and at Keck II on Mauna Kea in Hawaii. The system is designed to provide operational robustness matching the requirements of astronomical observation. All optical components before the highly nonlinear fibre are polarization maintaining fibre-based, so as to eliminate the effect of polarization drift on spectral broadening in the highly nonlinear fibre. Moreover, no temperature control is required at the two telescope facilities. As a result, the comb is able to maintain its frequency, bandwidth and intensity without the need to adjust any parameters. During a 5 day run at IRTF, the comb had zero failures and the intensity of individual comb teeth was measured to deviate less than 2 dB, including multiple power-off and on cycling of the optical continuum generation system (see Fig. 4b).

### Comb stability

As noted above, the frequency stability of the LR-EOFC is dominated by the stability of the reference laser frequency *f*_0_. We explored the use of two different commercially available lasers (Wavelength References) that were stabilized, respectively, to Doppler- and pressure-broadened transitions in acetylene (C_2_H_2_) at 1,542.4 nm, and in hydrogen cyanide (H^13^C^15^N) at 1,559.9 nm. We note that the spectroscopy related to the locking of the reference laser to the molecular resonances is done internally to the laser system, so that our experiments only assess the stability of these commercial off-the-shelf lasers. To assess the stability, the stabilized laser frequencies were measured relative to an Er:fibre-based self-referenced optical frequency comb^11^,^32^. Fibre-coupled light from a reference laser was combined into a common optical fibre with light from the Er:fibre comb. Then the heterodyne beat between a single-comb line and the line-stabilized reference was filtered, amplified and counted with a 10 s gate time using a frequency counter that was referenced to a hydrogen maser (see Fig. 3b). The Er:fibre comb was stabilized relative to the same hydrogen maser, such that the fractional frequency stability of the measurement was <2 × 10^−13^ at all averaging times. The drift of the hydrogen maser frequency is <1 × 10^−15^ per day, thereby providing a stable reference at levels corresponding to a RV uncertainty 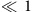 cm s^−1^. Thus, the frequency of the counted heterodyne beat accurately represents the fluctuations in the reference laser.

The series of 10 s measurements of the heterodyne beat was recorded over 20 days in 2013 for the case of the 1,542.4 nm laser and more than 7 days in 2014 for the case of the 1,559.9 nm laser, as shown in Fig. 3c. Gaps in the measurements near 11/31 and 6/4 are due to unlocking of the Er:fibre comb from the hydrogen maser reference. From these time series, we calculate the Allan deviation, which is a measure of the fractional frequency fluctuations (instability) of the reference laser as a function of averaging time. As seen in Fig. 3d, the instability of the 1,542.2 nm laser is <10^−9^ (30 cm s^−1^ RV, or corresponding to 200 kHz in frequency) at all averaging times greater than ∼30 s. The 1,559.9 nm laser is less stable, but provides a corresponding RV precision of <60 cm s^−1^ for averaging times greater than 20 s. This different instability was to be expected because of the difference in relative absorption line strength between the acetylene and HCN-stabilized lasers. In both cases, the stability improves with averaging time, although at a rate slower than predicted for white frequency noise. As an aside, we note that despite the lower stability of the 1,559.9 nm laser, this wavelength ultimately produced wider and flatter comb spectra owing to the better gain performance of the fibre amplifier used in this work. We did not explore the noise mechanisms that lead to the observed Allan deviation, as they arise from details of the spectroscopy internal to the commercial off-the-shelf laser, to which we did not have access.

Additional analysis included an estimate of the drift of the frequencies of the two reference lasers obtained by fitting a line to the full multi-day counter time series. From these linear fits, an upper limit of the drift over the given measurement period was determined to be <9 × 10^−12^ per day for the acetylene-referenced laser and <4 × 10^−11^ for the hydrogen cyanide-referenced laser. This corresponds to equivalent RV drifts of <0.27 and <1.2 cm s^−1^ per day for the two references. Finally, we attempted to place a bound on the repeatability of the 1,542.4 nm reference laser during re-locking and power cycling. Although only evaluated for a limited number of power cycles and re-locks, in all cases, we found that the laser frequency returned to its predetermined value within <100 kHz, or equivalently, with a RV precision of <15 cm s^−1^.

While these calibrations are sufficient for the few-day observations reported below, confidence in the longer term stability of the molecularly referenced continuous-wave lasers would be required for observations that could extend over many years. Likewise, frequency uncertainty of the molecular references should be examined. Properly addressing the potential frequency drifts on such a multi-year time scale would require a more thorough investigation of systematic frequency effects due to a variety of physical and operational parameters (for example, laser power, pressure, temperature and electronic offsets). Alternatively, narrower absorption features, as available in nonlinear Doppler-free saturation spectroscopy, could provide improved performance. For example, laboratory experiments have shown fractional frequency instability at the level of 10^−12^ and reproducibility of 1.5 × 10^−11^ for lasers locked to a Doppler-free transition in acetylene^33^. Most promising of all, self-referencing of an EOFC comb has been demonstrated recently^34^, enabling full stabilization of the frequency comb to a GPS-disciplined standard. This would eliminate the need for the reference laser to define *f*_0_, and thereby provide comb stability at the level of the GPS reference (for example, <10^−11^ or equivalently <0.3 cm s^−1^) on both long and short timescales.

### IRTF telescope demonstration

To demonstrate that the laser comb is portable, robust and easy-to-use as a wavelength calibration standard, we shipped the laser comb to the NASA IRTF. IRTF is a 3 m diameter infrared-optimized telescope located at the summit of Mauna Kea, Hawaii. The telescope is equipped with a cryogenic echelle spectrograph (CSHELL) operating from 1–5.4 μm. CSHELL is a cryogenic, near-infrared traditional slit-fed spectrograph, with a resolution^35^,^36^ of *R*∼*λ*/Δ*λ*=46,000 and it images an adjustable single ∼5-nm-wide order spectrum on a 256 × 256 InSb detector. We have modified the CSHELL spectrograph to permit the addition of a fibre acquisition unit for the injection of starlight and laser frequency comb light into a fibre array and focusing on the spectrograph entrance slit. A simple schematic of the fibre acquisition unit is shown in Fig. 2 and the details are described elsewhere^37^,^38^. Before the starlight reaches the CSHELL entrance slit, it can be switched to pass through an isotopic methane absorption gas cell to introduce a common optical path wavelength reference^38^. A pickoff mirror is next inserted into the beam to re-direct the near-infrared starlight to a fibre via a fibre-coupling lens. A dichroic window re-directs the visible light to a guide camera to maintain the position of the star on the entrance of the fibre tip. For the starlight, we made use of a specialized non-circular core multi-mode fibre, with a 50 × 100 μm rectangular core. These fibres ‘scramble' the near-field spatial modes of the fibre, so that the spectrograph is evenly illuminated by the output from the fibre, regardless of the alignment, focus or weather conditions of the starlight impinging upon the input to the fibre. We additionally made use of a dual-frequency agitator motor to vibrate the 10 m length of the fibre to provide additional mode mixing, distributing the starlight evenly between all modes. Finally, a lens and a second pickoff mirror are used to relay the output of the starlight from the fibre output back to the spectrograph entrance slit. A single-mode fibre carrying the laser comb is added next to the non-circular core fibre carrying the starlight. This was accomplished by replacing the output single-fibre SMA-fibre chuck with a custom three-dimensional printed V-groove array ferrule. This allowed us to send the light from both the star and frequency comb to the entrance slit of the CSHELL spectrograph when rotated in the same orientation as the slit.

Finally, the laser comb and associated electronics rack were set-up in the room temperature (∼±5 °C) control room. A 50 m length of single mode fibre was run from the control room to the telescope dome floor, and along the telescope mount to the CSHELL spectrograph to connect to the V-groove array and the fibre acquisition unit. The unpacking, set-up and integration of the comb fibre with CSHELL were straightforward, and required only 2 days working at an oxygen-deprived elevation of 14,000 feet in preparation for the observing run. Because the CSHELL spectrometer has a spectral window <5 nm, there was no effort made to generate spectrally flat combs. Comb lines are well resolved on CSHELL from 1,375 to 1,700 nm (Fig. 4a), with power adjusted by tunable optical attenuators to match the power of starlight and 6.7 pixels per comb line spacing at 1,670 nm wavelength. Also, comb line power was monitored (Fig. 4b) periodically during the observing run and was stable.

Three partial nights of CSHELL telescope time in September 2014 were used for this first on-sky demonstration of the laser comb. Unfortunately, the observing run was plagued by poor weather conditions, with 5–10 magnitudes of extinction because of clouds. Consequently, we observed the bright M2 II–III star *β* Peg (H=−2.1 mag), which is a pulsating variable star (*P*=43.3 days). Typical exposure times were 150 s, and multiple exposures were obtained in sequence.

The star was primarily observed at 1,670 nm, with and without the isotopic methane gas cell to provide a wavelength calibration comparison for the laser comb. Other wavelengths were also observed to demonstrate that the spectral grasp of the comb is much larger than the spectral grasp of the spectrograph itself. Given the low SNR (signal-to-noise ratio) on *β* Peg from the high extinction because of clouds and CSHELL's limited spectral grasp, the SNR of these data is inadequate to demonstrate that the comb is more stable than the gas cell, as shown above.

One critical aspect of demonstrating the usability of the comb for astrophysical spectrographs is the comb line spacing. As seen in Fig. 4a,c,d, the spectra clearly demonstrate that the individual comb lines are resolved with the CSHELL spectrograph without the need for additional line filtering^39^. Thus this comb operates at a frequency that is natively well-suited for astronomical applications with significantly less hardware complexity compared with ‘traditional' laser frequency combs.

### Keck telescope demonstration

We were able to use daytime access to the near-infrared cryogenic echelle spectrograph (NIRSPEC) on the Keck-II telescope^40^ to demonstrate our laser comb. NIRSPEC is a cross-dispersed echelle capable of covering a large fraction of the entire H-band in a single setting with a spectral resolution of R∼25,000. Observations were taken on 18 and 19 May 2015, with the comb set-up in the Keck-II control room in the same configuration as at the IRTF. The apparatus was reassembled after almost 8 months of storage from the time of the IRTF experiment and was fully operational within a few hours. The fibre output from the comb was routed through a cable wrap up to the Nasmyth platform where NIRSPEC is located. We injected the comb signal using a fibre feed into the integrating sphere at the input to the NIRSPEC calibration subsystem. While this arrangement did not allow for simultaneous stellar and comb observations, we were able to measure the comb lines simultaneously with the arc lamps normally used for wavelength calibration and to make hour-long tests of the stability of the NIRSPEC instrument at the sub-pixel level.

**Figure 5:**
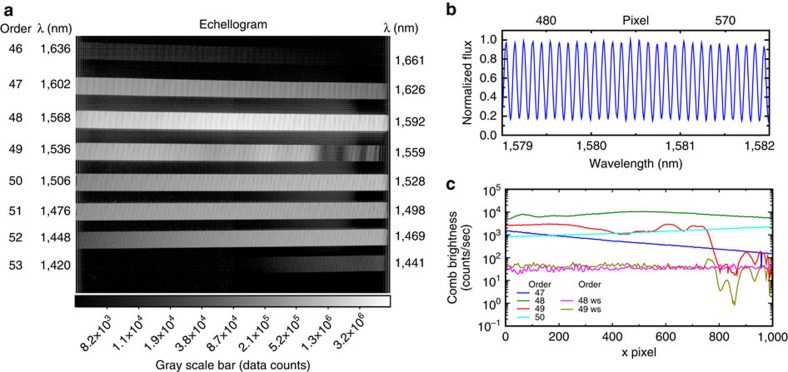
Data from testing at Keck II. (**a**) Reduced NIRSPEC image from echelle order 46–53, displaying the stabilized laser comb using the 1,559.9 nm reference laser. Line brightness represents data counts. (**b**) A portion of the extracted comb spectrum from order 48 is plotted versus wavelength. (**c**) Comb brightness envelope of orders 47–50 and orders 48 and 49 when flattened by a waveshaper (ws).

Figure 5a shows the laser comb illuminating more than six orders of the high-resolution echellogram. The echelle data were reduced in standard fashion, correcting for dark current and flat-field variations. Under this comb setting, a spectral grasp of ∼200 nm is covered, from 1,430 to 1,640 nm. A zoomed-in spectral extraction (Fig. 5b) shows that individual comb lines are well resolved at NIRSPEC's resolution and spaced approximately 4 pixels apart (0.1 nm), consistent with the higher resolution IRTF observations described above. The spectral intensity of the comb lines can be made more uniform with a flattening filter to allow constant illumination over the entire span. In this demonstration, we were also able to implement a programmable optical filter (Waveshaper 1000s) from 1,530 to 1,600 nm, greatly reducing comb intensity variation (Plots 48ws and 49ws in Fig. 5c). If desired, a customized filter could increase the bandwidth of the flattened regime to cover the entire comb span.

We used a series of 600 spectra taken over a ∼2 h time period to test the instrumental stability of NIRSPEC. Order 48, which had the highest SNR comb lines, was reduced following a standard procedure to correct for dark current and flat-field variations. Due to the quasi-Littrow configuration of the instrument, the slits appear tilted on the detector and the spectra have some curvature. We performed a spatial rectification using a flat-field image taken with a pinhole slit to mimic a bright compact object on the spectrum in order to account for this curvature. Wavelength calibration and spectral rectification to account for slit tilting were applied using the Ne, Kr, Ar and Xe arc lamps and the rectification procedure in the REDSPEC software written for NIRSPEC.

**Figure 6:**
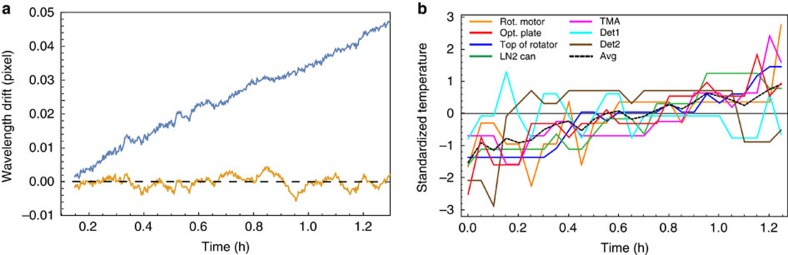
Measurement of wavelength and temperature drift on the Keck II NIRSPEC spectrometer. (**a**) The blue curve shows the drift in the pixel location of individual comb lines in order 48 as measured with the cross-correlation techniques described in the text. The yellow curve shows the residual shifts after de-correlating the effects of the internal NIRSPEC temperatures. (**b**) Five internal NIRSPEC temperatures are shown as a function of time. For ease of plotting, the individual temperatures have been standardized with respect to the means and s.d. of each sensor (Table 1). The black dashed curve shows the average of these standardized temperatures. The effect of the quantization of the temperature data at the 10 mK level (as recorded in the available telemetry) is evident in the individual temperature curves.

Instrumental stability was tested by performing a cross-correlation between the first comb spectrum in the 600 image series and each successive comb spectrum. The peak of the cross-correlation function corresponded to the drift, measured in pixels, between the images. Figure 6a demonstrates the power of the laser comb to provide a wavelength standard for the spectrometer. Over a period of roughly an hour the centroid of each comb line in Order 48 moved by about 0.05 pixel, equivalent to 0.0114 Å. By examining various internal NIRSPEC temperatures it is possible to show that this drift correlates to changes inside the instrument. Figure 6b shows changes in the temperatures measured at five different points within the instrument: the grating mechanism motor, an optical mounting plate, the top of the grating rotator mechanism, the base of the (unused) LN_2_ container and the three mirror anastigmat assembly^40^. At these locations the temperatures range from 50 to 75 K and have been standardized to fit onto a single plot: Θ_*i*_(*t*)=(*T*_*i*_(*t*)−<*T*>)/*σ*(*T*). Average values of each temperature are given in Table 1 and show drifts of order 15–35 mK over this 1 h period. In its present configuration NIRSPEC is cooled using a closed cycle refrigerator without active temperature control—only the detector temperature is maintained under closed cycle control to ∼1 mK.

Examination of the wavelength and temperature drifts in the two figures reveals an obvious correlation. A simple linear fit of the wavelength drift to the five standardized temperatures reduces the temperature-induced wavelength drifts from 0.05 pixel per hour to a near-constant value with a s.d. of *σ*=0.0017 pixel for a single-comb line (bottom curve in Fig. 6a). While other mechanical effects may manifest themselves in other or longer time series, this small data set indicates the power of the laser comb to stabilize the wavelength scale of the spectrometer. At the present spectral resolution of NIRSPEC, *R*∼25,000, and with over 240 comb lines in just this one order, we can set a limit on the velocity drift due to drifts within NIRSPEC of 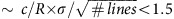 m s^−1^ where *c* is the speed of light.

Thus, operation with a laser comb covering over 200 nm with more than 2,000 lines in the H-band would allow much higher RV precision than is presently possible using, for example, atmospheric OH lines, as a wavelength standard. NIRSPEC's ultimate RV precision will depend on many factors, including the brightness of the star, NIRSPEC's spectral resolution (presently 25,000 but increasing to 37,500 after a planned upgrade) and the ability to stabilize the input stellar light against pointing drifts and line profile variations. We anticipate that in an exposure of 900 s NIRSPEC should be able to achieve an RV precision ∼1 m s^−1^ for stars brighter than H=7 mag and <3 m s^−1^ for a stars brighter than H<9 mag. A detailed discussion of the NIRSPEC error budget is beyond the scope of this paper, but a stable wavelength reference, observed simultaneously with the stellar spectrum, is critical to achieving this precision.

## Discussion

Many challenges remain to achieving the high precision RV capability needed for the study of exoplanets orbiting late M dwarfs, jitter-prone hotter G and K spectral types, or young stars exhibiting high levels of RV noise in the visible. Achieving adequate signal-to-noise on relatively faint stars requires a large spectral grasp on a high-resolution spectrometer on a large aperture telescope. Injecting both the laser comb and starlight into the spectrograph with a highly stable line spread function demands carefully designed interfaces between the comb light and starlight at the entrance to the spectrograph. Extracting the data from the spectrometer requires careful attention to flat-fielding and other detector features. Finally, reducing the extracted spectra to produce RV measurements at the required level of precision requires sophisticated modelling of complex stellar atmospheres and telluric atmospheric absorption. The research described here addresses only one of these steps, namely the generation of a highly stable wavelength standard in the near IR suitable for sub m s^−1^ RV measurements.

## Additional information

**How to cite this article:** Yi, X. *et al*. Demonstration of a near-IR line-referenced electro-optical laser frequency comb for precision radial velocity measurements in astronomy. *Nat. Commun*. 7:10436 doi: 10.1038/ncomms10436 (2016).

## Figures and Tables

**Table 1:** Internal NIRSPEC temperatures (K).

Rotator motor	54.944±0.015	Optics plate	52.887±0.023
Top of rotator	74.778±0.035	LN_2_ Can	53.663±0.021
TMA	53.866±0.022		

NIRSPEC, near-infrared cryogenic echelle spectrograph; TMA, three mirror anastigmat.

## References

[b1] Perryman M. The Exoplanet Handbook Cambridge University Press (2011).

[b2] Marcy G. & Howard A. The astrophysics of planetary systems: formation, structure, and dynamical evolution. in Proceedings IAU Symposium vol. 276, 3–12 (2011).

[b3] Kasting J. F., Whitmire D. P. & Reynolds R. T. Habitable zones around main sequence stars. Icarus 101, 108–128 (1993). 11536936 10.1006/icar.1993.1010

[b4] Pepe F., Ehrenreich D. & Meyer M. R. Instrumentation for the detection and characterization of exoplanets. Nature 513, 358–366 (2014). 25230658 10.1038/nature13784

[b5] Murphy M. . High-precision wavelength calibration of astronomical spectrographs with laser frequency combs. Mon. Not. R. Astron. Soc. 380, 839–847 (2007).

[b6] Osterman S. . Optical Engineering + Applications 66931G–66931GInternational Society for Optics and Photonics (2007).

[b7] Li C.-H. . A laser frequency comb that enables radial velocity measurements with a precision of 1 cm/s. Nature 452, 610–612 (2008). 18385734 10.1038/nature06854

[b8] Braje D., Kirchner M., Osterman S., Fortier T. & Diddams S. Astronomical spectrograph calibration with broad-spectrum frequency combs. Eur. Phys. J. D 48, 57–66 (2008).

[b9] Glenday A. G. . Operation of a broadband visible-wavelength astro-comb with a high-resolution astrophysi-cal spectrograph. Optica 2, 250–254 (2015).

[b10] Steinmetz T. . Laser frequency combs for astronomical observations. Science 321, 1335–1337 (2008). 18772434 10.1126/science.1161030

[b11] Yeas G. G. . Demonstration of on-sky calibration of astronomical spectra using a 25 Ghz near-IR laser frequency comb. Opt. Express 20, 6631–6643 (2012). 22418547 10.1364/OE.20.006631

[b12] Quinlan F., Yeas G., Osterman S. & Diddams S. A 12.5 Ghz-spaced optical frequency comb spanning> 400 nm for near-infrared astronomical spectrograph calibration. Rev. Sci. Instrum. 81, 063105 (2010). 20590223 10.1063/1.3436638

[b13] Jones D. J. . Carrier-envelope phase control of femtosecond mode-locked lasers and direct optical frequency synthesis. Science 288, 635–639 (2000). 10784441 10.1126/science.288.5466.635

[b14] Cundiff S. T. & Ye J. Colloquium: femtosecond optical frequency combs. Rev. Mod. Phys. 75, 325 (2003).

[b15] Diddams S. A. The evolving optical frequency comb [invited]. JOSA B 27, B51–B62 (2010).

[b16] Wilken T. . A spectrograph for exoplanet observations calibrated at the centimetre-per-second level. Nature 485, 611–614 (2012). 22660320 10.1038/nature11092

[b17] Wildi F., Pepe F., Chazelas B., Curto G. L. & Lovis C. SPIE Astronomical Telescopes I Instrumentation 77354X–77354XInternational Society for Optics and Photonics (2010).

[b18] Halverson S. . Development of fiber fabry-perot interferometers as stable near-infrared calibration sources for high resolution spectrographs. Publ. Astron. Soc. Pac. 126, 445–458 (2014).

[b19] Bauer F. F., Zechmeister M. & Reiners A. Calibrating echelle spectrographs with fabry-perot etalons, Astronomy & Astrophysics. 581, A117 (2015).

[b20] Kippenberg T. J., Holzwarth R. & Diddams S. Microresonator-based optical frequency combs. Science 332, 555–559 (2011). 21527707 10.1126/science.1193968

[b21] Del'Haye P., Arcizet O., Schliesser A., Holzwarth R. & Kippenberg T. J. Full stabilization of a microresonator-based optical frequency comb. Phys. Rev. Lett. 101, 053903 (2008). 18764394 10.1103/PhysRevLett.101.053903

[b22] Papp S. B. . Microresonator frequency comb optical clock. Optica 1, 10–14 (2014).

[b23] Suzuki S. . Nonlinear Optics NM3A–NM33Optical Society of America (2013).

[b24] Kotani T. . SPIE Astronomical Telescopes+ Instrumentation 914714–914714International Society for Optics and Photonics (2014).

[b25] Imai K., Kourogi M. & Ohtsu M. 30-thz span optical frequency comb generation by self-phase modulation in an optical fiber. IEEE J. Quantum Electron. 34, 54–60 (1998).

[b26] Fujiwara M., Kani J., Suzuki I. I., Araya K. & Teshima M. Flattened optical multicarrier generation of 12.5 Ghz spaced 256 channels based on sinusoidal amplitude and phase hybrid modulation. Electron. Lett. 37, 967–968 (2001).

[b27] Huang C.-B., Park S.-G., Leaird D. E. & Weiner A. M. Nonlinearly broadened phase-modulated continuous-wave laser frequency combs characterized using dpsk decoding. Opt. Express 16, 2520–2527 (2008). 18542332 10.1364/oe.16.002520

[b28] Morohashi I. . Widely repetition-tunable 200 fs pulse source using a mach-zehnder-modulator-based fiat comb generator and dispersion-flattened dispersion-decreasing fiber. Opt. Lett. 33, 1192–1194 (2008). 18516170 10.1364/ol.33.001192

[b29] Ishizawa A. . Phase-noise characteristics of a 25-ghz-spaced optical frequency comb based on a phase-and intensity-modulated laser. Opt. Express 21, 29186–29194 (2013). 24514470 10.1364/OE.21.029186

[b30] Dudley J. M., Genty G. & Coen S. Supercontinuum generation in photonic crystal fiber. Rev. Mod. Phys. 78, 1135 (2006).

[b31] Mori K. Supercontinuum lightwave source employing fabry-perot filter for generating optical carriers with high signal-to-noise ratio. Electron. Lett. 41, 975–976 (2005).

[b32] Ycas G., Osterman S. & Diddams S. Generation of a 660–2100, nm laser frequency comb based on an erbium fiber laser. Opt. Lett. 37, 2199–2201 (2012). 22739854 10.1364/OL.37.002199

[b33] Edwards C. S. . Absolute frequency measurement of a 1.5-*μ*m acetylene standard by use of a combined frequency chain and femtosecond comb. Opt. Lett. 29, 566–568 (2004). 15035472 10.1364/ol.29.000566

[b34] Beha K. . Self-referencing a continuous-wave laser with electro-optic modulation. Preprint at arXiv:1507.06344 (2015).

[b35] Greene T. P., Tokunaga A. T., Toomey D. W. & Carr J. B. in Optical Engineering and Photonics in Aerospace Sensing 313–324International Society for Optics and Photonics (1993).

[b36] Tokunaga A. T., Toomey D. W., Carr J. B., Hall D. N. & Epps H. W. Astronomy'90, Tucson AZ, 11-16 Feb 90, 131–143International Society for Optics and Photonics (1990).

[b37] Plavchan P. P. . SPIE Optical Engineering + Applications 88641J–88641JInternational Society for Optics and Photonics (2013).

[b38] Plavchan P. P. . SPIE Optical Engineering+ Applications 88640G–88640GInternational Society for Optics and Photonics (2013).

[b39] Osterman S. . EPJ Web of Conferences vol. 16, 02002(EDP Sciences (2011).

[b40] McLean I. S. . Astronomical Telescopes and Instrumentation 566–578International Society for Optics and Photonics (1998).

